# Effect of Temperature and Insect Infestation Levels on Oxygen Depletion in Hermetic Storage

**DOI:** 10.3390/insects14070621

**Published:** 2023-07-10

**Authors:** Trust Kasambala Donga, Dieudonné Baributsa

**Affiliations:** 1 Crop and Soil Sciences Department (CSSD), Bunda Campus, Lilongwe University of Agriculture and Natural Resources (LUANAR), P.O. Box 219, Lilongwe 207203, Malawi; tdonga@luanar.ac.mw; 2 Department of Entomology, Purdue University, 901 W. State St., W. Lafayette, IN 47907, USA

**Keywords:** oxygen consumption, storage pests, insect population, grain protection

## Abstract

**Simple Summary:**

We examined the effect of temperature and initial pest infestation levels on oxygen depletion during hermetic storage. Jars were filled with cowpea grains and infested with 25 or 50 cowpea weevils. The jars were then hermetically sealed and stored at 20, 30, or 40 °C for 30 days. The results showed that oxygen depletion during hermetic storage varied depending on the temperature and the level of insect infestation. This study revealed that the optimum temperature for effectively depleting oxygen to 5% or below, regardless of the infestation level, was 30 °C. Grain quality was maintained with minimal damage and losses. Only at 20 °C did a few adult insects survive hypoxia for 30 days, and some emerged 45 days later when the jars were exposed to normal oxygen conditions. Therefore, hermetic storage containers should remain closed for more than 30 days to minimize re-infestation risks in areas where the average ambient temperature rarely exceeds 23 °C.

**Abstract:**

Hermetic storage methods are effective at protecting grain against insect pests. Biotic and abiotic factors influence oxygen depletion during hermetic storage. We investigated the dual effects of temperature and initial pest infestation level on oxygen depletion during airtight storage. Glass jars filled with cowpea grain were infested (25 or 50 adult cowpea bruchids), then hermetically sealed and stored at 20, 30, or 40 °C for 30 days. Oxygen depletion, relative humidity, and temperature were monitored. Germination, grain moisture content, grain damage and weight loss, and adult emergence were assessed. Oxygen depletion varied by temperature and insect infestation level. However, 30 °C was the optimum temperature for oxygen depletion (reaching 5% or less in 10 days) regardless of insect infestation level. No changes were observed in germination and grain moisture content, minimal grain damage, or weight loss (<1%). Only at 20 °C were adult insects able to survive after 30 days and emerged 45 days post-treatment under normoxia. Therefore, hermetic storage containers should remain closed for more than 30 days to minimize re-infestation of grain in areas where average ambient temperatures rarely exceed 23 °C. Further research is needed to assess the effect of low temperatures on oxygen depletion and insect survival in hermetic storage beyond 30 days.

## 1. Introduction

Hermetic or airtight storage technologies are chemical-free methods used to protect grains against insect pests. Commonly used airtight storage methods include silos (e.g., metal and plastics) and hermetic bags such as Purdue Improved Crop Storage (PICS) [1]. Hermetic storage works by restricting gaseous exchange between the inside and outside of the container [2,3,4]. A reduction in oxygen level (hypoxia) and a corresponding increase in carbon dioxide gas within the hermetic container occurs due to oxidative metabolism primarily caused by insects [5]. Once the oxygen level within the airtight container has dropped to 5% or below, insects will stop feeding and developing, leading to death [5,6,7].

Maintaining a low-oxygen environment for an extended period is crucial to avoid residual insect infestation [8]. Several factors affect how quickly and how much oxygen is depleted in hermetic containers, including temperature and insect infestation level. Temperature influences insect oxygen consumption during hermetic storage [9]. In warmer regions such as West Africa, the oxygen level in hermetic (PICS) bags can decrease to below 10% within 10 days. However, in cooler areas such as East Africa, grain stored under similar conditions for the same duration or longer rarely experiences the same significant decrease in oxygen level [2,10,11,12]. The temperature in cooler regions leads to decreased metabolic activity, resulting in low oxygen consumption, while the contrary is seen at high temperatures [13,14,15,16].

Insects have a high metabolic rate, and their respiratory systems require oxygen for energy production. As a result, increased insect infestation levels can lead to accelerated oxygen consumption in their immediate surroundings. The effect of insect infestation level on oxygen consumption can be particularly pronounced in confined spaces, such as hermetic storage. Research has shown that the initial grain infestation level significantly impacts oxygen depletion during grain storage in hermetic systems [3,6]. Regardless of insect infestation level, airtight storage methods are highly effective in preserving grain against insect attacks at low or high infestation levels [17].

No study has examined the dual effects of initial pest infestation level and varying temperatures on oxygen depletion in hermetic storage systems. We conducted this study to assess the impact of temperature and insect infestation level on oxygen depletion, insect mortality, and adult resurgence. The findings from this study will help farmers improve grain protection using hermetic storage. We hypothesize that high temperature (above 20 °C) and insect infestation play a critical role in accelerating oxygen depletion and hence limit insect survival and resurgence.

## 2. Materials and Methods

### 2.1. Insect Rearing and Grain Preparation

*Callosobrocus maculatus* adults were reared on cowpea grain (variety #8046, The Wax Company, LLC, Amory, MS, USA) in a Conviron Environmental Chamber (C710, Winnipeg, MB, Canada) at 25 ± 1 °C, relative humidity (RH) of 40 ± 5%, and photoperiod of 12:12 (L:D). One month before the experiment, the *C. maculatus* adults were put on cowpea grain and removed after allowing them to lay eggs for 24 h. The first cohort of emerging adults was removed using the number 5 Endecott sieve. After 48 h, freshly emerged adults (within two days of emergence) were used for the experiment. Cowpea grains were frozen at −20 °C for 14 days (for disinfestation) and thawed at room temperature for two days before the beginning of the experiment.

### 2.2. Experimental Design

The experiment was conducted from September to November 2021 in the Postharvest Innovation for Crop Storage Laboratory, Department of Entomology at Purdue University, West Lafayette, Indiana. Thirty-six 1000 mL glass jars (Ball Corporation, Broomfield, CO, USA) were used for the experiment, and each contained 693.19 ± 2.78 g of cowpea grain. The treatments were a combination of jars infested with 25 or 50 insects stored at 20, 30, or 40 °C. The experiment was conducted for 30 days and ran twice (two-run). Three Caron insect growth chambers (Model 6025, Marietta, OH, USA) were set at a fixed temperature (20, 30, or 40 °C). Relative humidity was maintained at 50% in all chambers. After the experiment, grain was kept under normoxia for 45 days at 25 °C to assess progeny.

### 2.3. Data Collection

#### 2.3.1. Oxygen Measurement

To measure the oxygen concentration, a fluorescent yellow Oxydot (Industrial Physics, Boston, MA, USA) was glued to the inner wall of each jar. One oxygen reading was recorded per replicate at the onset of the experiment, every 24 h for the first 15 days, and on day 30 using an OxySense 5250i oxygen reader (Industrial Physics, Boston, MA, USA). The device was equipped with a fiber optic oxygen reader pen that measured the change in intensity and fluorescent characteristics of the Oxydots.

#### 2.3.2. Temperature and Relative Humidity

Data loggers (EL-USB-2, Lascar Electronics Inc., Erie, PA, USA) were used to record the temperature and RH every hour. A data logger was kept inside each growth chamber and in two replications of each treatment. At the end of the experiment, data were downloaded from the loggers as MS Excel files for processing.

#### 2.3.3. Grain Quality Assessments

Grain quality was assessed at the beginning and the end of the experiment using several parameters. The number of live *C. maculatus* adults was estimated using the whole sample. However, the number of larvae, eggs, damaged grains (*Nd*), and undamaged grains (*Nu*) was estimated using random samples of 100 seeds from each treatment replication. The weight of the damaged (*D*) and undamaged grains (*U*) was then determined using an analytical balance with a precision of 0.01 mg. The percent insect grain damage was calculated as (*Nd/(Nd + Nu)*) × 100 [11]. The count and weight method were used to calculate percentage grain weight loss using the formula: % weight loss = (*(U × Nd) − (D × Nu)*)/*U (Nu + Nd*) × 100 [18].To assess the number of larvae, the 100 cowpea seeds of each replicate were soaked in distilled water for 24 h before being split to expose the larva. The moisture content of the cowpea grains was measured before and after the experiment using a DICKEY-John mini-GAC^®^ plus grain moisture tester (Auburn, IL, USA). To assess germination, a random sample of 25 grains per replicate was used [19]. The grains were disinfested in a 4.5% bleach solution for 5 min and then rinsed three times (30 s in each rinse) with distilled water. Each grain sample was wrapped with wet paper towels, placed in a 9 cm diameter Petri dish, and stored at ambient room temperature (25 °C). Moisture was sustained by misting grains with distilled water every day. Once every day for seven days, the number of grains with at least part of the radicle breaking through the seed coat was counted as germinated. Percentage germination was calculated using the formula: % Germination = (Total number of seeds germinated/Total number of seeds) × 100.

### 2.4. Statistical Analysis

Data were first entered into MS Excel to facilitate the calculation of grain weight loss, grain damage, moisture content, and germination rates. Statistical analyses were performed in SPSS version 28 (IBM SPSS Statistics, Chicago, IL, USA). The effects of the experimental run, time, temperature, insect infestation level, and their interactions on oxygen consumption were determined using linear mixed models. Trend analysis was performed to develop oxygen consumption models at different temperatures. A significant difference was considered when the coefficient of the interaction term for the treatment factors (temperature and insect infestation level) reached statistical significance. Treatment means were separated using Bonferroni adjustment at alpha level 0.05.

## 3. Results

### 3.1. Oxygen Measurement

**Figure 1:**
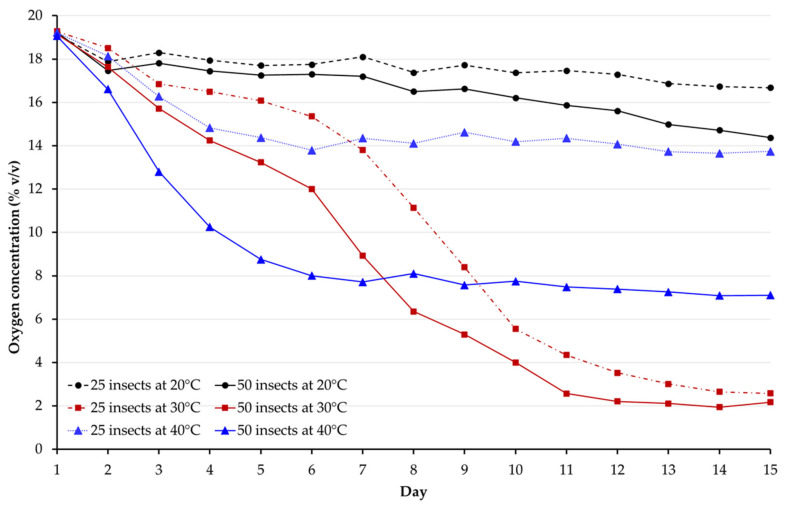
Oxygen levels in hermetic jars containing grains infested with 25 or 50 *C. maculatus* adults and stored at 20, 30, or 40 °C for 15 days.

The oxygen concentration level declined following unique patterns contingent on the original insect pest infestation level and temperature (Figure 1). Within five days, the oxygen concentration had dropped to below 10% in the treatment with the highest infestation level (50 insects) and stored at the highest temperature, 40 °C (Table 1). After 10 days, the oxygen concentration was ≤5% for treatments stored at 30 °C irrespective of pest infestation level.

The linear mixed model results, without considering time as a factor, indicated that the experimental runs had no significant effect on oxygen depletion (F = 1.074, *p =* 0.301). Hence, the data for the two experimental runs were combined for analysis. Then, the linear mixed model was performed again by considering the time factor. The coefficient of interaction for time and treatment was not significant (F = 1.357, *p =* 0.099). However, the interaction of insect infestation level × temperature (F = 48.578, *p =* 0.001), insect infestation level × time (F = 3.007, *p =* 0.001), and temperature × time (F = 39.379, *p =* 0.001) were significant. This meant that the insect infestation level and temperature were potential significant predictors of oxygen depletion during grain storage in hermetic jars.

The fixed effects of temperature, insect infestation level, and time on oxygen depletion as generated by the linear mixed model are presented in Table 2. All fixed effects were statistically significant, with *p*-values smaller than 0.05. The fixed effects of the insect infestation level on oxygen consumption were positive, while that of temperature was negative. There was a 4.34 percentage point increase in the oxygen concentration with a 25-unit decrease in insect infestation level (from 50 to 25 *C. maculatus* adults). There was a 4.91 percentage point drop in oxygen concentration with a 10-unit decrease in temperature from 40 to 30 °C. The negative effects of temperature and insect infestation level on oxygen depletion tended to get stronger over time, as indicated by the negative signs of their interactions. At a 25-insect infestation level, there was a 3.92 percentage point drop in oxygen concentration with a 10-unit decrease in temperature from 40 to 30 °C.

The effects of the initial insect infestation level and temperature in the hermetic containers may be generalized using the equations shown in Table 3. Based on these equations, oxygen depletion in the sealed jars at 20 °C followed a linear function. However, at 30 and 40 °C, oxygen depletion in the hermetic jars followed a polynomial function.

### 3.2. Temperature and Relative Humidity

**Figure 2:**
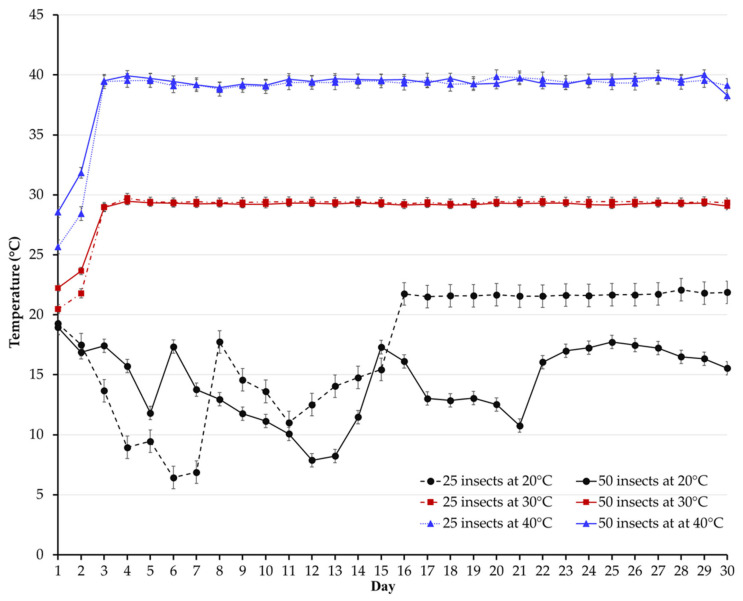
Temporal fluctuations in temperature (°C) inside hermetic jars containing grains infested with 25 or 50 *C. maculatus* adults and stored at 20, 30, or 40 °C for 30 days.

The experiment was conducted under a controlled temperature (20, 30, and 40 °C) and relative humidity (50%). During the first 12 days of storage, in the chamber set at 20 °C, the temperature inside the jars fluctuated significantly between 6 and 23 °C (Figure 2). This issue was due to insect growth chamber dysfunction. The temperature in the other treatments remained within the set limits. The relative humidity remained relatively steady in all treatments (data not shown).

### 3.3. Grain Moisture Content and Percentage Germination

The initial grain moisture content and the germination rate were 12% and 99.8%, respectively (data not shown). At the end of the experiment (after 30 days), the moisture content averaged 12%, and the germination rate was 98.5%. No significant changes in the grain moisture content (F = 2.329, *p =* 0.062) or germination rates (F = 0.718, *p* = 0.614) were observed.

### 3.4. Insect Occurrence, Grain Damage, and Weight Loss

The initial assessment indicated no pest infestation (no eggs, adults, grain damage, or weight loss). At the end of the experiment, live *C. maculatus* adults were observed only in those treatments kept at 20 °C (F = 11.97, *p =* 0.003) (Table 4). Significant differences were observed in the number of eggs laid per insect (F = 9.53, *p =* 0.007) among the treatments. However, this did not translate into a significantly higher number of developed larvae (F = 1.00, *p =* 0.332). Grain damage was only observed for the treatment with 50 insects at 30 °C (1.97%), with significant differences compared to the other treatments (F = 23.83, *p =* 0.000). However, weight loss was below 1% (data not shown), and no significant differences were observed among the treatments (F = 1.00, *p =* 0.332). After 45 days of incubation post-storage under normoxia, adults emerged in the treatments previously stored at 20 °C (Table 4).

## 4. Discussion

This study evaluated the effects of temperature and insect infestation level on oxygen depletion during grain storage in hermetic jars. Mixed linear models indicated that temperature and insect infestation level are predictors of oxygen depletion during grain storage in airtight containers. At each temperature, oxygen depletion was lower at an infestation level of 25 compared to 50 insects, though statistical differences varied over time.

Oxygen depletion slowed at the lowest temperature (i.e., at 20 °C). Although the temperature fluctuated considerably in the surrounding environment (6–23 °C), the effects of low temperature on insect physiology and oxygen consumption align with findings from previous studies. The slight decrease in oxygen at 20 °C can be primarily attributed to a reduction in overall metabolic activity [13,14,16,20]. On the contrary, when the temperature increased to 30 °C, oxygen depletion occurred rapidly, dropping to 10% within just nine days. The same trend was seen at 40 °C but only at a 50-insect infestation level. As in this study, the optimum temperature for the development of *C. maculatus* was 30 °C [15,20,21]. We can reasonably say that adult *C. maculatus*, which live for approximately 10–14 days, remained metabolically active at 30 °C until the end of their lifespan. The earlier flattening of the curve for treatments stored at 40 °C can be attributed to several factors. First, during the initial hours of the experiment, the insects exhibited significantly higher oxygen consumption at 40 °C, regardless of the level of infestation. Second, a temperature of 40 °C is known to have detrimental effects on the development of *C. maculatus* [15,22]. Research has shown that *C. maculatus* adults’ longevity is shortened when exposed to 40 °C [20].

Insects are ectothermic organisms, and their metabolic rate strongly depends on temperature. The impact of temperature on oxygen depletion suggests that in regions where the average temperature hovers at or below 20 °C during grain storage, the oxygen concentration can take several weeks or even months to decrease to a critical level necessary to eliminate insect infestation. On the contrary, in areas where the mean temperature exceeds 30 °C during storage, insects exhibit intense oxygen consumption, resulting in a rapid decline in oxygen levels. These phenomena have been observed in studies conducted on the use of hermetic bags in sub-Saharan Africa. For instance, it took approximately 90 days for the oxygen level to reach 5% in Kenya, where temperatures during grain storage varied between 22 and 27 °C [11]. Conversely, only one to six days were required for the oxygen level within hermetic bags to drop below 5% in Niger, where the storage temperature ranged from 25 to 41 °C [2].

Furthermore, this study suggests that the level of initial insect infestation of the grain influences the rate of oxygen depletion in the hermetic storage system. The rate of oxygen decline was accelerated in treatments with a higher insect infestation level. Similar results have been reported in previous studies where the oxygen depletion rate was proportional to the initial insect infestation level [6,17,23]. However, the impact of the initial infestation on oxygen depletion was less pronounced when grain was stored at 30 °C, which is optimal for cowpea development [15,20,21]. At 30 °C, oxygen fell to 5% or below within 10 days for infestation levels of 25 and 50 insects, and no significant differences were observed between them.

No adult insects survived in cowpea grain stored in hermetic jars kept at 30 and 40 °C for 30 days. However, a few larvae and adults remained alive in cowpea grain in airtight jars kept at 20 °C. A few adult insects survived for two weeks beyond their lifespan. Our results are consistent with earlier studies that showed very low survival of insects during hermetic storage [4,6,7,24]. Extended insect survival beyond their lifespan can be attributed to changes in insect behavior, specifically their transition to diapause in response to unfavorable conditions such as hypoxia and low-temperature environments [25,26]. This phenomenon delays aging in adult insects, resulting in extended longevity. The survival of insects in these low-oxygen environments is rare and poorly understood [3,4,6,23], requiring further investigation.

Temperatures affected the insect activity, impacting oxygen consumption and progeny development 45 days post-treatment under normoxia. A high temperature of 40 °C shortened the longevity of the adults and killed the eggs and larvae, hence leading to no offspring. Our findings are corroborated by other studies that showed no progeny development when insects were exposed to 40 °C or above [15,20]. At 30 °C, oxygen depletion to below 5% in nine days affected adults, eggs, and larvae, leading to no progeny development. These findings have the potential for commercial applications. Investigating the effect of a high temperature of 30 °C or above on oxygen consumption and/or early death of insects inside hermetic bags in warehouses could provide some insights. However, further research is warranted to explore this concept further. At 20 °C, fewer eggs were laid, and few adults emerged after 45 days post-treatment under normoxia. The development of progeny at 20 °C can be explained by the survival of eggs due to a moderate temperature and supply of oxygen, which was above 14% and 7% after 15 and 30 days, respectively. Previous studies have indicated that insect exposure to hypoxia or low temperatures decreases mating, oviposition, and progeny development [3,7,25].

## 5. Conclusions

This study demonstrated how temperature and pest infestation level affect oxygen consumption under hermetic storage, grain quality, and subsequent progeny development. Infested grain hermetically stored at a high temperature of 40 °C led to a quick death of adult insects and eggs, resulting in no progeny development. At 30 °C, oxygen depletion reached 5% or below within 10 days, leading to no survival of adult insects or eggs and no progeny development 45 days post-treatment. Exploring the effect of high temperatures on oxygen consumption and/or the early death of insects inside hermetic bags in warehouses could be helpful for commercial applications. At 20 °C, there was minimal oxygen consumption, resulting in the survival of a few adult insects and the emergence of some adult insects 45 days post-treatment. The optimum temperature for oxygen depletion was 30 °C regardless of insect infestation. We recommend that infested grain stored hermetically in places where the average ambient temperature rarely exceeds 23 °C remain under closed conditions for longer than 30 days to minimize re-infestation. Further research is needed to understand how low temperatures affect oxygen consumption and insect survival during hermetic storage beyond 30 days.

## Figures and Tables

**Table 1:** Analysis of variance of the residual oxygen level after 5, 10, 15, and 30 days in hermetic jars containing grains infested with 25 or 50 *C. maculatus* adults and stored at 20, 30, or 40 °C for 30 days.

Treatment	Residual Oxygen Level (Percentage, Mean ± SEM)			
	Day 5	Day 10	Day 15	Day 30
25 insects at 20 °C	18 ± 0.200a *	17 ± 0.149a	16 ± 0.354a	7 ± 1.091b
50 insects at 20 °C	17 ± 0.302a	16 ± 0.575a	14 ± 1.002a	7 ± 1.060b
25 insects at 30 °C	16 ± 0.402a	5 ± 0.806c	3 ± 0.766d	1 ± 0.469c
50 insects at 30 °C	12 ± 1.096b	3 ± 0.752c	2 ± 0.532d	3 ± 1.966c
25 insects at 40 °C	13 ± 0.291b	12 ± 2.169b	11 ± 2.538b	9 ± 1.396a
50 insects at 40 °C	8 ± 1.037c	7 ± 1.186c	7 ± 1.089c	6 ± 1.475ab
F (*p*-value)	38.58 (<0.001)	69.61 (<0.001)	88.58 (<0.001)	4.06 (0.004)

* All data are mean ± standard error of means (SEM). Means in the same column followed by the same letters are not significantly different at *p* = 0.05.

**Table 2:** Linear mixed model estimates of fixed effects of temperature, insect infestation level, and time on oxygen depletion.

Parameter *	Estimate	SE ^a^	*t*	*p*-Value	LCI ^b^	UCI ^c^
Intercept	6.84	0.66	10.34	<0.001	5.54	8.13
25 insects	4.34	1.07	4.07	<0.001	2.25	6.43
50 insects	0	0	.	.	.	.
20 °C	7.46	0.97	7.71	<0.001	5.56	9.36
30 °C	−4.91	0.94	−5.26	<0.001	−6.75	−3.08
40 °C	0	0	.	.	.	.
25 insects at 30 °C	−3.92	1.42	−2.77	0.006	−6.70	−1.14
25 insects at 40 °C	0	0	.	.	.	.
Initially (time = 0)	12.23	0.94	13.09	<0.001	10.40	14.07
25 insects initially	−4.18	1.42	−2.95	0.003	−6.97	−1.40
20 °C initially	−7.30	1.35	−5.43	<.001	−9.94	−4.66
25 insects at 30 °C initially	3.89	1.94	2.01	0.045	0.085	7.70

* Degree of freedom for all parameters = 640. ^a^ Standard error; ^b^ lower confidence interval; ^c^ upper confidence interval.

**Table 3:** Relationship between oxygen concentration (y) at any time (x) in hermetic jars containing grains infested with 25 or 50 *C. maculatus* adults and stored at 20, 30, or 40 °C for 30 days.

Treatment	Equation *	*R*² Value
25 insects at 20 °C	y = −0.148x + 18.77	0.897
50 insects at 20 °C	y = −0.3137x + 19.024	0.961
25 insects at 30 °C	y = 0.009x^3^ − 0.2204x^2^ + 0.1119x + 19.271	0.979
50 insects at 30 °C	y = 0.0029x^3^ − 0.0055x^2^ − 1.9252x + 21.602	0.986
25 insects at 40 °C	y = −0.0045x^3^ + 0.1611x^2^ − 1.8512x + 20.687	0.923
50 insects at 40 °C	y = −0.0087x^3^ + 0.3296x^2^ − 3.9746x + 22.408	0.968

* Where y is the oxygen concentration level at time x.

**Table 4:** Grain infestation after 30 days of storage in hermetic jars containing grains initially infested with 25 or 50 *C. maculatus* adults and stored at 20, 30, or 40 °C, and adult emergence 45 days post-treatment after exposure to normoxia at 25 °C. Initial values were zero for eggs, larvae, and adults before artificially infesting jars containing cowpea grains.

	Grain Infestation Levels after 30 Days			Adult Emergence 45 Days Post-Treatment
Treatments	Eggs	Larvae	Adults	
25 insects at 20 °C	13.5 ± 5.98c	0 ± 0.47a	1.4 ± 0.72ab	1.25 ± 0.75b
50 insects at 20 °C	17.6 ± 6.93c	0 ± 0.54a	4.7 ± 0.83a	5.86 ± 1.78a
25 insects at 30 °C	30.8 ± 5.99b	0 ± 0.47a	0 ± 0.00b	0 ± 0.00c
50 insects at 30 °C	48.0 ± 5.99a	1.1 ± 0.47a	0 ± 0.00b	0 ± 0.00c
25 insects at 40 °C	14.0 ± 5.99c	0 ± 0.47a	0 ± 0.00b	0 ± 0.00c
50 insects at 40 °C	30.5 ± 5.99b	0 ± 0.47a	0 ± 0.00b	0 ± 0.00c
F (*p*-value)	9.53 (0.007)	1.00 (0.332)	11.97 (0.003)	11.16 (0.004)

All data are mean ± standard error of means (SEM). Means in the same column followed by the same letters are not significantly different at *p* = 0.05.

## Data Availability

Raw data are not publicly available.

## References

[1] Baributsa D., Njoroge A.W. (2020). The use and profitability of hermetic technologies for grain storage among smallholder farmers in eastern Kenya. J. Stored Prod. Res..

[2] Baoua I.B., Margam V., Amadou L., Murdock L.L. (2012). Performance of triple bagging hermetic technology for postharvest storage of cowpea grain in Niger. J. Stored Prod. Res..

[3] Kharel K., Mason L.J., Murdock L.L., Baributsa D. (2019). Efficacy of hypoxia against *Tribolium castaneum* (Coleoptera: Tenebrionidae) throughout ontogeny. J. Econ. Entomol..

[4] Kandel P., Scharf M.E., Mason L.J., Baributsa D. (2021). Effect of hypoxia on the lethal mortality time of adult *Sitophilus oryzae* L.. Insects.

[5] Murdock L.L., Margam V., Baoua I., Balfe S., Shade R.E. (2012). Death by desiccation: Effects of hermetic storage on cowpea bruchids. J. Stored Prod. Res..

[6] Njoroge A.W., Mankin R.W., Smith B., Baributsa D. (2019). Stored-product effects of hypoxia on acoustic activity of two stored-product pests, adult emergence, and grain quality. J. Econ. Entomol..

[7] Yan Y., Williams S.B., Baributsa D., Murdock L.L. (2016). Hypoxia treatment of *Callosobruchus maculatus* females and its effects on reproductive output and development of progeny following exposure. Insects.

[8] Quellhorst H.E. (2018). Oxgen Consumption by Grain Storage Pests in Relation to Hermetic Storage Systems and Evaluation of Postharvest Management Practices by Smallholder Farmers in Haiti. Doctoral Dissertation.

[9] Donahaye E.J., Navarro S., Rindner M., Azrieli A. (1996). The combined influence of temperature and modified atmospheres on *Tribolium castaneum* (Herbst) (coleoptera: Tenebrionidae). J. Stored Prod. Res..

[10] Njoroge A.W., Affognon H.D., Mutungi C.M., Manono J., Lamuka P.O., Murdock L.L. (2014). Triple bag hermetic storage delivers a lethal punch to *Prostephanus truncatus* (Horn) (Coleoptera: Bostrichidae) in stored maize. J. Stored Prod. Res..

[11] Mutungi C.M., Affognon H., Njoroge A.W., Baributsa D., Murdock L.L. (2014). Storage of mung bean (*Vigna radiata* [L.] Wilczek) and pigeonpea grains (*Cajanus cajan* [L.] Millsp) in hermetic triple-layer bags stops losses caused by *Callosobruchus maculatus* (F.) (Coleoptera: Bruchidae). J. Stored Prod. Res..

[12] Baoua I.B., Bakoye O., Amadou L., Murdock L.L., Baributsa D. (2018). Performance of PICS bags under extreme conditions in the sahel zone of Niger. J. Stored Prod. Res..

[13] Neven L.G. (2000). Physiological responses of insects to heat. Postharvest Biol. Technol..

[14] Renault D., Hervant F., Vernon P. (2003). Effect of food shortage and temperature on oxygen consumption in the lesser mealworm, *Alphitobius diaperinus* (Panzer) (Coleoptera: Tenebrionidae). Physiol. Entomol..

[15] Lale N.E., Vidal S. (2003). Effect of constant temperature and humidity on oviposition and development of *Callosobruchus maculatus* (F.) and *Callosobruchus subinnotatus* (Pic) on bambara groundnut, *Vigna subterranea* (L.) Verdcourt. J. Stored Prod. Res..

[16] Lalouette L., Williams C.M., Hervant F., Sinclair B.J., Renault D. (2011). Metabolic rate and oxidative stress in insects exposed to low temperature thermal fluctuations. Comp. Biochem. Physiol. Part A Mol. Integr. Physiol..

[17] Baoua I.B., Amadou L., Baributsa D., Murdock L.L. (2014). Triple bag hermetic technology for post-harvest preservation of Bambara groundnut (*Vigna subterranea* (L.) Verdc.). J. Stored Prod. Res..

[18] Boxall R. (1986). A critical review of the methodology for assessing farm level grain losses after harvest. Int. Biodeterior..

[19] Williams S.B., Murdock L.L., Baributsa D. (2017). Sorghum seed storage in Purdue Improved Crop Storage (PICS) bags and improvised containers. J. Stored Prod. Res..

[20] Osman M., Mahmoud M., Mohamed K. (2015). Susceptibility of Certain Pulse Grains to *Callosobruchus maculatus* (F.) (Bruchidae: Coleoptera), and Influence of Temperature on Its Biological Attributes. J. Appl. Plant Prot..

[21] Chandrakantha J., Mathavan S. (1986). Changes in developmental rates and biomass energy in *Callosobruchus maculatus* (F.) (Coleoptera: Bruchidae) reared on different foods and temperatures. J. Stored Prod. Res..

[22] Loganathan M., Jayas D.S., Fields P.G., White N.D.G. (2011). Low and high temperatures for the control of cowpea beetle, *Callosobruchus maculatus* (F.) (coleoptera: Bruchidae) in chickpeas. J. Stored Prod. Res..

[23] Njoroge A.W.W., Mankin R.W.W., Smith B.W.W., Baributsa D. (2017). Effects of Hermetic Storage on Adult *Sitophilus oryzae* L. (Coleoptera: Curculionidae) Acoustic Activity Patterns and Mortality. J. Econ. Entomol..

[24] Singano C.D., Mvumi B.M., Stathers T.E. (2019). Effectiveness of grain storage facilities and protectants in controlling stored-maize insect pests in a climate-risk prone area of Shire Valley, Southern Malawi. J. Stored Prod. Res..

[25] Ramadan M.M., Abdel-Hady A.A.A., Guedes R.N.C., Hashem A.S. (2020). Low temperature shock and chill-coma consequences for the red flour beetle (*Tribolium castaneum*) and the rice weevil (*Sitophilus oryzae*). J. Therm. Biol..

[26] Hutfilz C. (2022). Endocrine Regulation of Lifespan in Insect Diapause. Front. Physiol..

